# Discerning novel splice junctions derived from RNA-seq alignment: a deep learning approach

**DOI:** 10.1186/s12864-018-5350-1

**Published:** 2018-12-27

**Authors:** Yi Zhang, Xinan Liu, James MacLeod, Jinze Liu

**Affiliations:** 1 Department of Computer Science, University of Kentucky, Lexington, KY 40506 USA; 2 Department of Veterinary Science, University of Kentucky, Lexington, KY 40506 USA

**Keywords:** Deep learning, Splice junction, RNA-seq, Exon splicing

## Abstract

**Background:**

Exon splicing is a regulated cellular process in the transcription of protein-coding genes. Technological advancements and cost reductions in RNA sequencing have made quantitative and qualitative assessments of the transcriptome both possible and widely available. RNA-seq provides unprecedented resolution to identify gene structures and resolve the diversity of splicing variants. However, currently available ab initio aligners are vulnerable to spurious alignments due to random sequence matches and sample-reference genome discordance. As a consequence, a significant set of false positive exon junction predictions would be introduced, which will further confuse downstream analyses of splice variant discovery and abundance estimation.

**Results:**

In this work, we present a deep learning based splice junction sequence classifier, named DeepSplice, which employs convolutional neural networks to classify candidate splice junctions. We show (I) DeepSplice outperforms state-of-the-art methods for splice site classification when applied to the popular benchmark dataset HS3D, (II) DeepSplice shows high accuracy for splice junction classification with GENCODE annotation, and (III) the application of DeepSplice to classify putative splice junctions generated by Rail-RNA alignment of 21,504 human RNA-seq data significantly reduces 43 million candidates into around 3 million highly confident novel splice junctions.

**Conclusions:**

A model inferred from the sequences of annotated exon junctions that can then classify splice junctions derived from primary RNA-seq data has been implemented. The performance of the model was evaluated and compared through comprehensive benchmarking and testing, indicating a reliable performance and gross usability for classifying novel splice junctions derived from RNA-seq alignment.

**Electronic supplementary material:**

The online version of this article (10.1186/s12864-018-5350-1) contains supplementary material, which is available to authorized users.

## Background

Technological improvements, reduced cost, and accessibility of RNA sequencing technologies have provided unprecedented visibility of the transcriptome through the deep sequencing of all mRNA transcripts present in a sample. Through analyses of mRNA-seq data, researchers now believe that 92–94% of mammalian protein-coding genes undergo alternative splicing, with roughly 86% of these containing a minor transcript isoform frequency of at least 15% in certain cell types, developmental time points, physiological states, or other conditions [1]. This is an 87–89% increase from 40 years ago when alternative exon structures from a single gene locus were first introduced and it was believed that only around 5% of genes in higher eukaryotes undergo alternative splicing [2].

The approach to defining exon junctions from RNA-seq data utilizes the subset of reads that have a gapped alignment to the reference genome. These reads can be aligned to two or more exons, indicating that there exist junctions joining adjacent exons. Whereas some mapping strategies [3–6] require pre-defined structural annotation of exon coordinates, more recently developed algorithms [7–11] can conduct ab initio alignment, which means that they do not rely on the existence of predetermined gene structure annotation and can potentially identify novel splice junctions between exons by the evidence of spliced alignments.

The accurate prediction of exon junctions is essential for defining gene structures and mRNA transcript variants. Splicing must be absolutely precise because the deletion or addition of even a single nucleotide at the splice junction would throw the subsequent three-base codon translation of the RNA out of frame [12]. However, novel splice junctions predicted by read alignments are not totally reliable, since the possibility of randomly mapping a short read up to 150 bases to the large reference genome is high [13], especially when gapped alignments with short anchoring sequences are permitted. In a recent report by Nellore et al. [14] that investigated splicing variation, 21,504 RNA-seq samples from the Sequenced Read Archive (SRA) were aligned to the human hg19 reference genome with Rail-RNA [15], identifying 42 million putative splice junctions in total. This value is 125 times the number of total annotated splice junctions in humans, making it impossible to admit that all of them actually exist. False positive splice junctions may lead to false edges in splice graphs, significantly increasing the complexity of the graphical structures [16]. Consequentially, this will impact the accuracy of splice variant inference algorithms as they often start from splice graphs derived from RNA-seq alignment [17].

Conventional strategies designed to filter out false positive exon splice junctions depend primarily on two properties: (1) the number and the diversity of reads mapped to the given splice junction [13]; and/or (2) the number of independent samples in which the specific exon splice junction is identified [13, 18]. In general, higher read support and sample reoccurrence rate both enlarge the likelihood of being a true splice junction. These criteria have a positive correlation with the number of read alignments, which are dependent on the sampling depth of the particular sample. Exact thresholds are difficult to set due to varying sampling depth across samples. Additionally, due to both sequencing and alignment errors, a splice junction with both high read support and high sample reoccurrence may still be the result of systematic bias. In contrast, a splice junction that exists in a transcript with relatively low expression may still be functionally important [19]. Thus, further classification of putative splice junctions revealed by RNA-seq data is still necessary but remains a challenging issue.

Since the 1980s, a number of bioinformatic approaches have been developed for splice site prediction. Neural networks [20–22], support vector machines [23–25], hidden Markov model [26–28], deep Boltzmann machines [29] and discriminant analysis [30, 31] have been applied to recognize splice sites in the reference genome of many given species. Neural networks, support vector machines and deep Boltzmann machines learn the complex features of neighborhoods surrounding the consensus dinucleotide AG/GT by a non-linear transformation. Hidden Markov models estimate position specific probabilities of splice sites by computing the likelihoods of candidate signal sequences. The discriminant analysis uses several statistical measures to evaluate the presence of specific nucleotides, recognizing splice sites without explicitly determining the probability distributions [28]. However, all these work treat donor and acceptor sites as independent events, failing to leverage the inherent relationships between the donor and acceptor during splicing.

In this paper, we develop a deep neural network-based approach to the classification of potential splice junctions. Our method is applicable to both splice site prediction and splice junction classification. First, instead of treating donor or acceptor splice sites individually, our method models the donor and acceptor splice sites as a functional pair. Thus, it is capable of capturing the remote relationships between features in both donor and acceptor sites that determine the splicing. Additionally, flanking subsequences from both exonic and intronic sides of the donor and acceptor splice sites will be used for learning and prediction, making it possible to understand the contribution of both coding and non-coding genomic sequences to the splicing. Our approach does not rely on sequencing read support or frequency of occurrence derived from experimental RNA-seq data sets, thus can be applied as an independent evidence for splice junction validation. Our experiments demonstrate that DeepSplice outperforms other state-of-the-art approaches [28, 32–36] when tested against a benchmarking dataset, *Homo sapiens* Splice Sites Database (HS3D), using a variety of evaluation metrics. Trained on an older version of the GENCODE project gene annotation data [37], we show that our algorithm can predict the newly annotated splice junctions with high accuracy and performs better than splice site-based approach. The application of DeepSplice to further classify putative intropolis human splice junction data by Nellore et al. [14] is able to eliminate around 83% unannotated splice junctions. We discover that the combinational information from the functional pairing of donor and acceptor sites facilitates the recognition of splice junctions and demonstrate from large amounts of sequencing data that non-coding genomic sequences contribute much more than coding sequences to the location of splice junctions [25, 38].

## Results

We first applied our approach to a benchmark dataset HS3D [39] and compared the performance with other state-of-the-art approaches for donor and acceptor splice site classification. We then evaluated DeepSplice’s performance by classifying annotated splice junctions from GENCODE gene annotation data [37]. Deep Taylor decomposition [40] was then applied for further interpretation of base level contribution of flanking splice sequence. Finally, we applied DeepSplice to intropolis [14], a newly published splice junction database with 42,882,032 splice junctions derived from 21,504 samples. The detailed results are described below. Supplementary results are available in Additional file 1.

### DeepSplice outperforms state-of-the-art splice site prediction method

We utilized HS3D [39] (*Homo sapiens* Splice Sites Data set, http://www.sci.unisannio.it/docenti/rampone/), a popular benchmark for measuring the quality of splice site classification methods. HS3D includes introns, exons and splice site sequences extracted from GeneBank Rel. 123. The splice site sequences in HS3D are with the length of 140 nucleotides. There are 2796 (2880) true donor (acceptor) splice sites and 271,937 (329,374) false donor (acceptor) splice sites which all contain conserved GT (AG) dinucleotides. We constructed the 1:10 data set, which contains all the true splice sites and 27,960 (28,800) randomly selected false donor (acceptor) splice sites. Binary classifications were conducted to identify the actual splice sites on donor and acceptor splice site data separately.

DeepSplice was trained on donor and acceptor splice site sequences separately in order to compare with state-of-the-art approaches of splice site classification. The exact same number of training and testing splice site sequences from HS3D were used for all approaches. Table 1 summarizes the classification accuracies on the 1:10 data set by 10-fold cross-validation. To measure the quality of the classification results, we employed sensitivity, specificity, and *Q*^9^ which is the global accuracy measure calculated from both sensitivity and specificity scores. Since the published splice site classification methods do not provide public tools for training and testing, the results of SVM + B [32], MM1-SVM [28], DM-SVM [33], MEM [34] and LVMM2 [35] were obtained from [33, 35]. As shown in Table 1, DeepSplice outperforms other methods in both sensitivity and specificity for both donor and acceptor splice site classification. For donor splice sites, there is a 95% likelihood that the confidence interval [0.0581, 0.0633] covers the true classification error of DeepSplice on the testing data. For acceptor splice sites, there is a 95% likelihood that the confidence interval [0.0814, 0.0872] covers the true classification error of DeepSplice on the testing data.

**Table 1:** Evaluation of DeepSplice and state-of-the-art approaches for donor (acceptor) site classification on HS3D data set

	Donor			Acceptor		
	Sensitivity	Specificity	Q^9^	Sensitivity	Specificity	Q^9^
LS-GKM	0.8679	0.8516	0.8595	0.8403	0.8319	0.8361
SVM + B	0.9406	0.9067	0.9212	0.9066	0.8797	0.8920
MM1-SVM	0.9256	0.9244	0.9247	0.8993	0.8869	0.8926
DM-SVM	0.9469	0.9339	0.9399	0.9215	0.9073	0.9136
MEM	0.9324	0.9275	0.9295	0.9153	0.8843	0.8978
LVMM2	0.9424	0.9242	0.9323	0.9122	0.8970	0.9039
DeepSplice	0.9571	0.9376	0.9465	0.9337	0.9139	0.9232

**Fig. 1:**
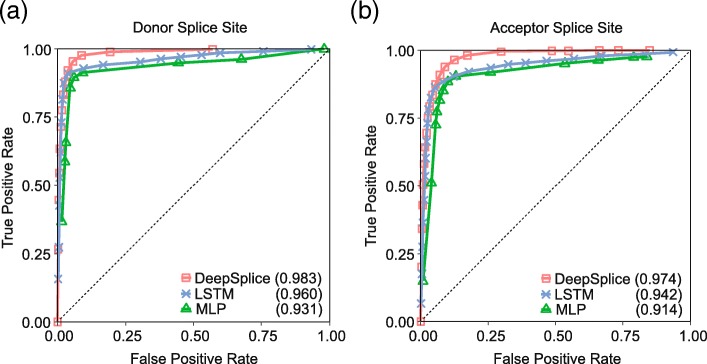
The ROC curves of DeepSplice, multilayer perceptron network (MLP) and long short-term memory network (LSTM) for (**a**) donor splice site and (**b**) acceptor splice site classification on the HS3D data set by 10-fold cross-validation. DeepSplice with convolutional neural network exceeds the other deep learning architectures, achieving an auROC score of 0.983 (0.974) on donor (acceptor) splice site classification

To deduce the most suitable architecture for learning the patterns in splice site/junction sequences, we then compared DeepSplice against two other prominent types of neural networks, multilayer perceptron network and long short-term memory network, in terms of classifying HS3D data set by 10-fold cross-validation. As shown in Fig. 1, DeepSplice with convolutional neural network exceeds the other architectures, achieving an auROC score of 0.983 (0.974) on donor (acceptor) splice site classification and an auPRC score of 0.863 (0.800) on donor (acceptor) splice site classification. LSTM achieved an auROC score of 0.960 (0.942) on donor (acceptor) splice site classification and an auPRC score of 0.803 (0.721) on donor (acceptor) splice site classification. MLP achieved an auROC score of 0.931 (0.914) on donor (acceptor) splice site classification and an auPRC score of 0.650 (0.559) on donor (acceptor) splice site classification. In general, convolutional neural network is a well-studied architecture, which outperforms other deep learning architectures in almost all kinds of applications currently [41]. Even for speech recognition, convolutional neural networks recently beat recurrent neural networks. In our application, convolutional layers efficiently learned the complex information of nucleotide neighborhoods.

### DeepSplice predicts newly annotated splice junctions with high accuracy

Next, we evaluated the accuracy of DeepSplice in terms of splice junction classification. To achieve this, we trained DeepSplice using splice junctions extracted from the GENCODE annotation version 3c, and then tested the model on newly annotated splice junctions in the GENCODE annotation version 19. All GENCODE splice junctions used for training and testing are experimental validated by RT-PCR amplification. The training set contains 521,512 splice junctions, and the testing set contains 106,786 splice junctions. In both training and testing sets, half of the splice junctions are annotated, and the rest are false splice junctions randomly sampled [42] from human reference genome (GRCh37/hg19).

**Fig. 2:**
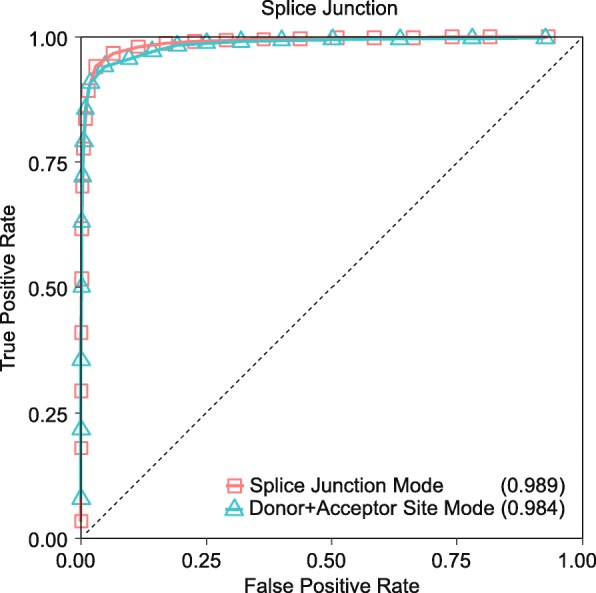
The ROC curves of DeepSplice Splice Junction Mode and Donor+Acceptor Site Mode for splice junction classification on the GENCODE data set. DeepSplice Splice Junction Mode achieves a higher auROC score of 0.989

We trained the first model by feeding the 521,512 training splice junction sequences to DeepSplice for a binary classification, splice junctions or not. In the meantime, we trained two other models separately by feeding the donor (acceptor) splice site sequences extracted from the 521,512 training splice junction sequences to DeepSplice for a binary classification, donor (acceptor) splice sites or not. This experiment was designed to determine whether making use of paired combinational information of donor and acceptor splice sites from a splice junction, instead of classifying donor or acceptor splice site individually, would ameliorate the quality of splice junction classification. In the first mode (Splice Junction Mode), the input splice junction sequences were with the length of 120 nucleotides, reflecting 30 nucleotides of upstream and downstream nucleotides for both donor and acceptor splice site. In the second mode (Donor+Acceptor Site Mode), the input splice junction sequences were split into two substrings with the length of 60 nucleotides and then fed to donor (acceptor) splice site classification model separately. For the second mode, we defined that the probability of a splice junction being classified as positive is the product of the probability of its donor splice site being classified as positive and the probability of its acceptor splice site being classified as positive, considering the two splice site classification events are statistically independent [43]. Figure 2 shows the ROC curves of the two modes. Splice Junction Mode achieved an auPRC score of 0.990, 0.987 for Donor+Acceptor Site Mode. Table 2 summarizes sensitivity, specificity, accuracy, and F1 score on the 106,786 testing splice junction sequences. Donor+Acceptor Site Mode acquires a higher specificity; however, Splice Junction Mode significantly outperforms Donor+Acceptor Site Mode in terms of sensitivity, accuracy, F1 score, auROC score, and auPRC score with substantially higher scores. In total, Splice Junction Mode predicted 50,340 out of 53,393 newly annotated splice junctions, which covered 9806 genes, 98.01% of all newly annotated genes. Donor+Acceptor Site Mode detected 39,067 splice junctions from 9185 genes. There is a 95% likelihood that the confidence interval [0.0432, 0.0456] covers the true classification error of DeepSplice on the testing splice junctions. These results indicate that the proposal splice junction classification in DeepSplice achieves high accuracy in identifying novel splice junctions in large data sets than conventional splice site classification.

**Table 2:** Classification performance evaluation of different DeepSplice modes on GENCODE data set

		Sensitivity	Specificity	Accuracy	F1 score
Splice site classification	Donor	0.917	0.897	0.907	0.908
	Acceptor	0.873	0.913	0.893	0.891
Splice junction classification	Splice Junction Mode	0.943	0.968	0.956	0.955
	Donor + Acceptor Site Mode	0.732	0.997	0.864	0.844

### Interpretation of sequence features captured by DeepSplice

There are highly conserved segments on splice junctions between exons and introns which help in the prediction of splice junctions by computational methods and decipher biological signals of splice junctions. We next further interpret which nucleotides contribute to the splicing process. This is achieved by the quantification of the contribution of nucleotides in splice junction sequences to the classification process using deep Taylor decomposition [40].

**Fig. 3:**
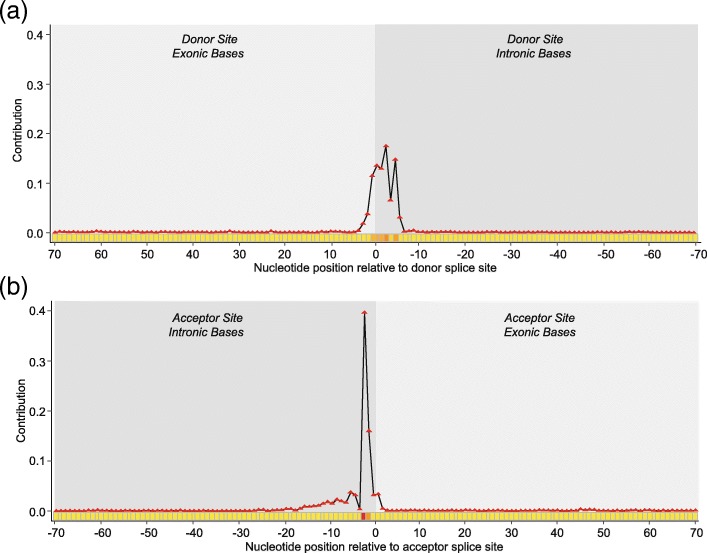
Visualization of the contribution of nucleotides in the flanking splice sequences to the final decision function of DeepSplice on the HS3D dataset for (**a**) donor splice site and (**b**) acceptor splice site classification. For both donor and acceptor site classifiers, intronic bases close to GT-AG di-nucleotides achieve the most importance in the classifiers. In general, intron sequences carry more discriminative information than exon sequences

**Fig. 4:**
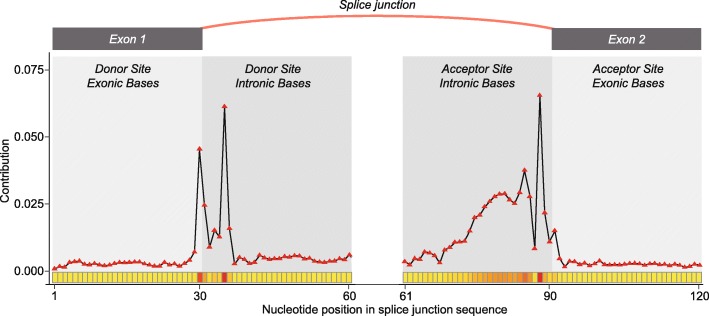
Visualization of the contribution of nucleotides in the flanking splice sequences to the final decision function of DeepSplice on the GENCODE dataset for splice junction classification. The nucleotides in the proximity of a splice junction have the highest impact on the classification outcome. As observed in the splice site classifiers, the contribution distribution of nucleotides in the flanking splice sequences indicates that intron nucleotides carry more discriminative information than exon nucleotides

DeepSplice employs convolutional neural network with two convolutional layers. In the convolutional layer, we defined filters with a shape of 3 × 1, which means filters scan the input sequence with a window size of 3 to learn the information of nucleotide neighborhoods. DeepSplice fundamentally is not using a single base but rather 3-mers or subsequences of length 3 as its features. Then deep Taylor decomposition runs a backward pass on the convolutional neural network to sign contributions. The contribution score of each single base in DeepSplice reflects the aggregated importance of the three 3-mers it belongs to. We first used deep Taylor decomposition to decompose cross-validation results of the HS3D dataset in terms of input splice site sequences. For nucleotides in the testing splice site sequences, scores were assigned to present their contribution. We obtained a graphical representation from which it is possible to judge which region in the splice site sequences is of importance. Figure 3 shows the contribution of nucleotides to the final decision function of DeepSplice. In general, intron sequences carry more discriminative information than exon sequences in this analysis. We then applied deep Taylor decomposition to the results of splice junction classification with the GENCODE data set. Figure 4 shows the contribution distribution of nucleotides in the testing splice junction sequences. Regions of increased importance in splice junction classification are consistent with the result from splice site classification.

### DeepSplice classification of intropolis

The intropolis v1 database [14] contains a large number of putative junctions found across 21,504 human RNA-seq samples in the Sequence Read Archive (SRA) from spliced read alignments to hg19 with Rail-RNA [15]. There are 42,882,032 putative splice junctions in total, including 18,856,578 canonical splice junctions containing flanking string GT-AG, 24,025,454 semi-canonical splice junctions containing flanking string AT-AC or GC-AG [44], and no non-canonical splice junctions which are not allowed by Rail-RNA. Table 3 lists the number of splice junctions in each category separated by the number of reoccurrence in samples and total read support across all samples in four scales: (a) equal to 1 {1}, (b) more than 1 and no greater than 10 (1, 10], (c) more than 10 and no greater than 1000 (10, 1000] and (d) more than 1000 (1000, +∞). As listed in Table 3, for our analysis, we only retain splice junctions in intropolis that are supported by more than one sample, followed by the filtering of false splice junction sequences due to repetitive sequences. After this pre-processing, 5,277,046 splice junctions were left for further classification.

**Table 3:** Distribution of splice junctions from intropolis given the reoccurrence in samples and total read support

Splice junction number		Reoccurrence in samples			
		{1}	(1, 10]	(10, 1000]	(1000, +∞)
Total reads	{1}	23 M	–	–	–
	(1, 10]	3331 K	11 M	–	–
	(10, 1000]	91 K	936 K	3301 K	–
	(1000, +∞)	38	187	124 K	305 K

“M” stands for “million”

“K” stands for “thousand”

**Fig. 5:**
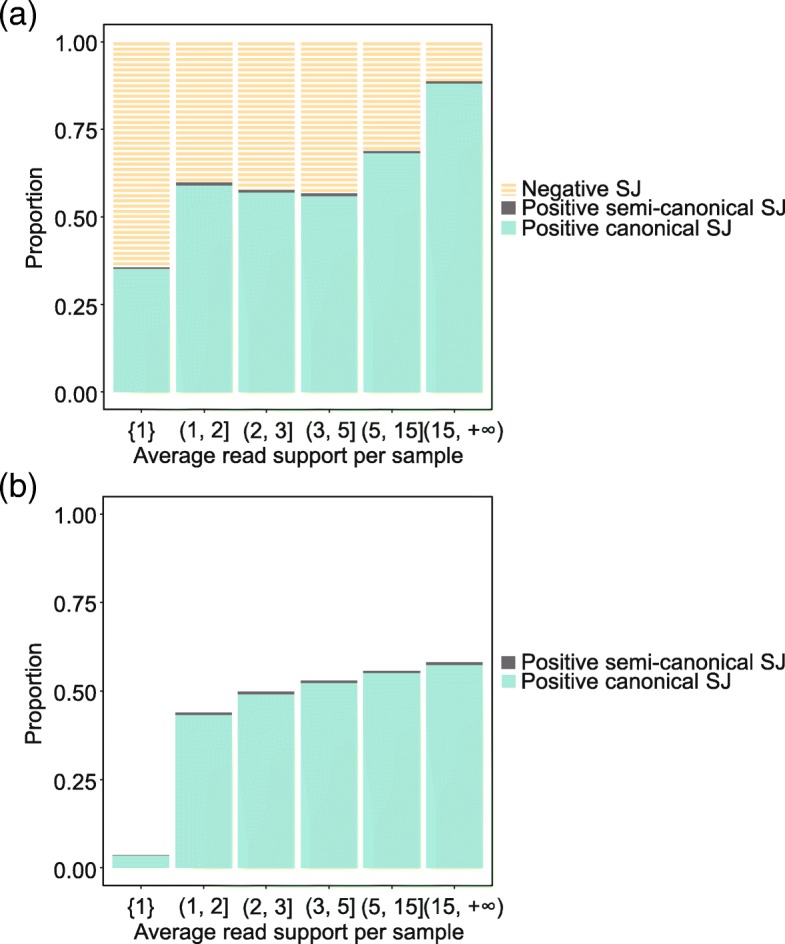
Positive splice junctions tend to have high read support and contain the canonical flanking string. **a** Discrete proportions of negatives, positive semi-canonical splice junctions and positive canonical splice junctions from the classification results, given the average read support per sample. Splice junctions with average read support per sample more than 15 achieve a positive rate of around 88%. In contrast, for splice junctions with average read support per sample no more than 1, only 36% are identified as positive. There is a significant rise in the probability to obtain a positive splice junction with the increase of the average read support per sample. Around 99% positive splice junctions contain the canonical flanking string. **b** Cumulative proportions of positive semi-canonical and canonical splice junctions with the increase of the average read support per sample

The DeepSplice model was trained on 812,967 splice junctions including (1) 291,030 annotated splice junctions from GENCODE annotation version 19, (2) 271,937 false splice junctions generated from the HS3D data set, and (3) 250,000 randomly selected semi-canonical splice junctions with only one read support from intropolis. Overall, DeepSplice classified 3,063,698 splice junctions as positive. Figure 5(a) lists the proportions of positive canonical splice junctions, positive semi-canonical splice junctions and negatives from the classification results at different levels of average read support per sample. Splice junctions with average read support per sample more than 15 achieve a positive rate around 88%. In contrast, for splice junctions with average read support per sample no more than 1, only 36% are identified as positives. There is a significant rise in the probability to obtain a positive splice junction with the increase of the average read support per sample. Around 99% positive splice junctions contain the canonical flanking string. Figure 5(b) illustrates the proportions of positive semi-canonical and canonical splice junctions cumulatively with the increase of the average read support per sample.

**Fig. 6:**
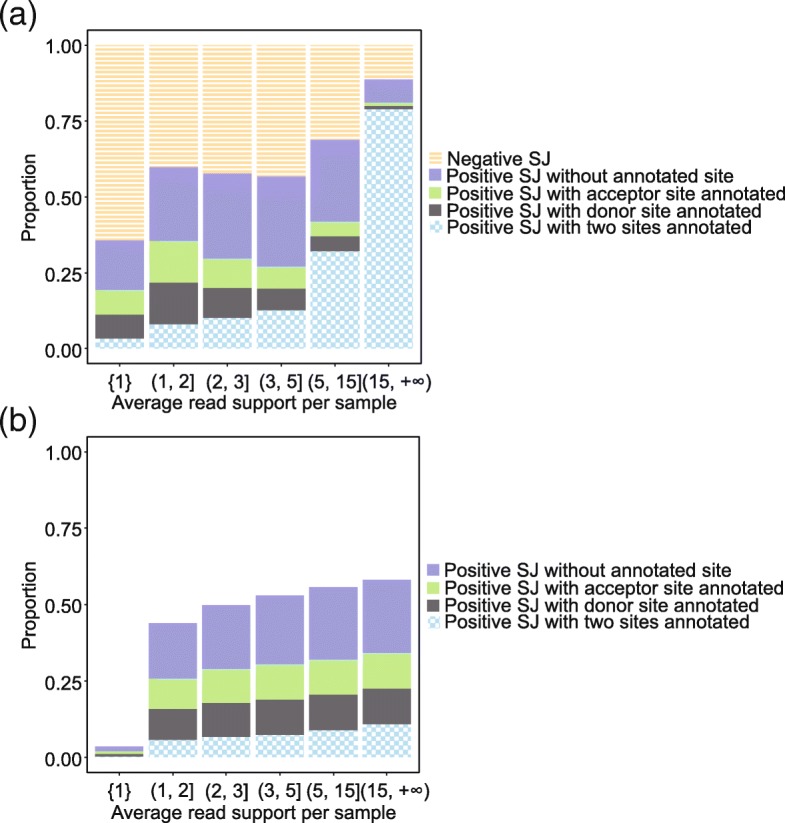
Positive splice junctions tend to have both donor and acceptor sites annotated. **a** Discrete proportions of negatives, positive splice junctions without annotated site, positive splice junctions with acceptor site annotated, positive splice junctions with donor site annotated and positive splice junctions with two sides annotated, given the average read support per sample. 97% of splice junctions with both sites annotated are classified as positives, while only 39% with both sites being novel are positive. Splice junctions connecting annotated splice sites also tend to be associated with higher read coverage. **b** Cumulative proportions of positive splice junctions in each category with the increase of the average read support per sample

**Fig. 7:**
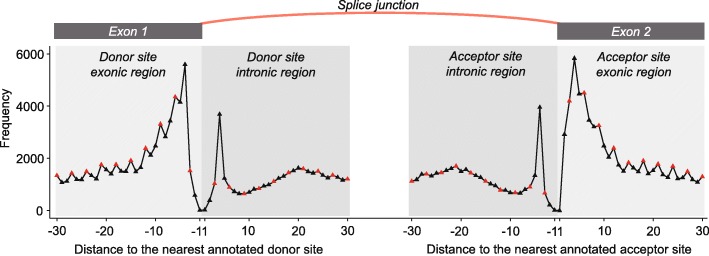
Splice sites which maintain the coding frame of the exon are observed more often than those which disrupt frame. Positive splice junctions in intropolis near known protein-coding junctions show a periodic pattern. For each donor (acceptor) site in the positive splice junctions, we calculated its distance to the nearest annotated donor (acceptor) site, and then counted the frequency for each position. The red points denote positions that are a multiple of three base pairs from the major splice form, and the black points those that are not

To further clarify characteristics of the positives, we categorized splice junctions in intropolis based on annotated splice sites in GENCODE annotation: (1) splice junctions with both splice sites annotated, (2) splice junctions with the donor splice site annotated, (3) splice junctions with the acceptor splice site annotated, and (4) splice junctions with neither the donor nor acceptor splice sites annotated. Figure 6(a) shows the discrete proportions of negatives and positive splice junctions in each category above, given the average read support per sample. Results indicate that 97% of splice junctions with both sites annotated are classified as positives, while only 39% with both sites being novel are positive. Splice junctions connecting annotated splice sites also tend to be associated with higher read coverage. Figure 6(b) illustrates the proportions of positive splice junctions in each category cumulatively with the increase of the average read support per sample. Figure 7 shows positive splice junctions in intropolis near known protein-coding junctions show a periodic pattern, such that splice sites which maintain the coding frame of the exon are observed more often than those which disrupt frame. This observation recapitulates patterns seen in studies of noisy splicing [19].

## Discussion

Even though splice junctions with high read support and/or high reoccurrence are more likely to be classified as real, a significant portion of relatively low-expressed splice junctions also carry true splicing signals. DeepSplice does not rely on sequencing read support, frequency of occurrence, or sequencing read length derived from experimental RNA-seq data sets, thus can be applied as an independent evidence for splice junction validation. The accumulation of RNA-seq data especially in different cell types, tissues and disease conditions will further consolidate the cell type-specificity and tissue-specificity of some of these junctions and their corresponding isoforms. DeepSplice may provide the first round of filtering of RNA-seq derived splice junctions for further structural validation, and studies that assess functional annotation of these splice junctions are warranted. DeepSplice could also extend its functionality to discriminate splice junctions that are highly or lowly supported by gene expression evidence and try to figure out what sequence patterns associate to this difference in future. For each input candidate splice junction, DeepSplice outputs a probability of being true, and the probability can be used as an input feature to the studies for learning the tissue-regulated splicing code [45] and the splicing in human tissues with a wide range of known diseases [46].

It is also well known that splicing can be changed due to mutations around the splice sites. Future studies that use subject-specific genomic sequences instead of reference genome sequences may further improve the accuracy of the DeepSplice model and classification performance. Additionally, DeepSplice can be further extended to the prediction of non-canonical splicing [47] that existing annotation has not captured, including not only exonic but also splicing involving Alu elements, small exons, and recursive splicing. Besides the classification of linear junctions, the identification of non-linear splice junctions, such as circRNA junctions will also expand the functionality of DeepSplice.

## Conclusions

Employing deep convolutional neural network, we develop DeepSplice, a model inferred from the sequences of annotated exon junctions that can then classify splice junctions derived from primary RNA-seq data, which can be applied to all species with sufficient transcript annotation to use as training data. Results demonstrate that DeepSplice outperforms the state-of-the-art splice site classification tools in terms of both classification accuracy and computational efficiency. Our findings further indicate that valuable information is present in the nucleotide sequence local to the splice junction, data that conventional splice site prediction techniques discard. Nucleotide representations learned from the input sequences are meaningful and improve accuracy. The major application of DeepSplice is the classification of splice junctions rather than individual donor or acceptor sites. For learning on large datasets of putative splice junctions, DeepSplice is orders of magnitude faster than the best performing existing alternatives, which becomes increasingly common considering the tremendous amount of new RNA-seq data being generated.

## Methods

**Fig. 8:**
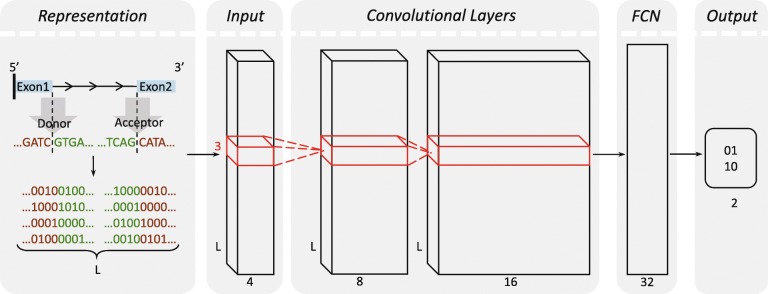
Visualization of splice junction sequence representation and deep convolutional neural network in DeepSplice. Each sequence is converted into a tensor through one-hot encoding in the pre-processing of the sequence representation. The tensor is fed as original input to the deep convolutional neural network, which contains one input layer, two convolutional layers, one fully connected layer (FCN) and one output layer. The convolutional neural network transforms the nucleotide signal in splice junction sequences to the final label of class

DeepSplice employs a convolutional neural network (CNN, or ConvNet) to understand sequence features that characterize real splice junctions. The overall architecture of DeepSplice is shown in Fig. 8. In the supervised training step, CNN learns features that help to differentiate actual splice junctions from fake ones. In the inference step, the trained model uses the genomic sequence of the candidate splice junction and predicts the probability of it being a real splice junction. Deep Taylor decomposition [40] of the CNN is used to explain to what extent each nucleotide in the candidate splice junction has contributed to the inference.

### Splice junction representation

A splice junction sequence is represented by four subsequences, the upstream exonic subsequence and downstream intronic subsequence at the donor site, and the upstream intronic subsequence and downstream exonic subsequence at the acceptor site, as shown in Fig. 8. Each subsequence has the length of 30, which is believed to be optimal for splice site/junction prediction [19, 22, 26, 27, 48]. Nucleotides in each sequence are represented through one-hot encoding, in which A, C, G, T and N are encoded by [0, 0, 0, 1] [0, 0, 1, 0, 0, 1, 0, 0] [1, 0, 0, 0] and [0.25, 0.25, 0.25, 0.25] respectively. In the proposed encoding system, the orthonormal sparse encoding is used for the four definite values (A, C, G and T) as it has been used widely in the numerical representations of biological sequences [49]. But for the ambiguous base N, instead of disregarding it or giving it the same importance as the definite values, the probability is used.

Each splice junction sequence is transformed into a 3-dimensional tensor of shape [height, width, channels]. The first dimension ‘height’ is equal to one, and the second dimension ‘width’ indexes the sequence length, that is, the number of nucleotides in the sequence, and the third dimension ‘channels’ indexes the type of nucleotide. The tensors are fed as input to deep convolutional neural networks for downstream processing.

### Deep convolutional neural network

DeepSplice contains a multi-layer feedforward neural network. We stack one input layer, two convolutional layers, one fully connected layer, and one output layer. The whole network architecture can be written as follows:

1 $$ Label\ of\ class={f}_{fcn}\left({f}_{conv2}\Big({f}_{conv1}\left( Sequence\ nucleotide\ signal\right)\right). $$

In this way, the convolutional neural network transforms the nucleotide signal in splice junction sequences to the final label of class as shown in Fig. 8.

In the first convolutional layer, the convolution will compute 8 features over the input tensor which represents splice junction sequence, which results in 8 feature maps of the input tensor. In order to reason the complex nonlinearity between inputs and outputs, we further stack the second convolutional layer computing 16 features over 8 feature maps from the first convolutional layer. In the convolutional layers, the filters have size 3 × 1. During the forward pass, we slide each filter along the splice junction sequence and compute dot products between the filter and the input tensor. As we slide the filter over the input splice junction sequence we will produce feature maps that give the responses of that filter at every spatial position. After two convolutional layers, the features are presented in 16 tensors. The output of the second convolutional layer is taken by a fully connected layer with 32 feature maps for high-level reasoning. The fully connected layer is followed by the output layer indicating the final label of class. In the neural network, all parameters are learned during training to minimize a loss function which captures the difference between the true labels of class and predicted values.

Training the network follows the standard backpropagation and optimizes the loss function using Adam [50]. Advance deep learning techniques L2 regularization [51], dropout [52] and mini-batch gradient descent [53] are deployed to regularize the network to prevent over-fitting and to accelerate the training process.

In the reference step, testing splice junction sequences transformed by one-hot encoding are fed to the learned network for a binary classification, which outputs the predicted label of the class, true or false splice junction.

### Deep Taylor decomposition of deep convolutional neural network

**Fig. 9:**
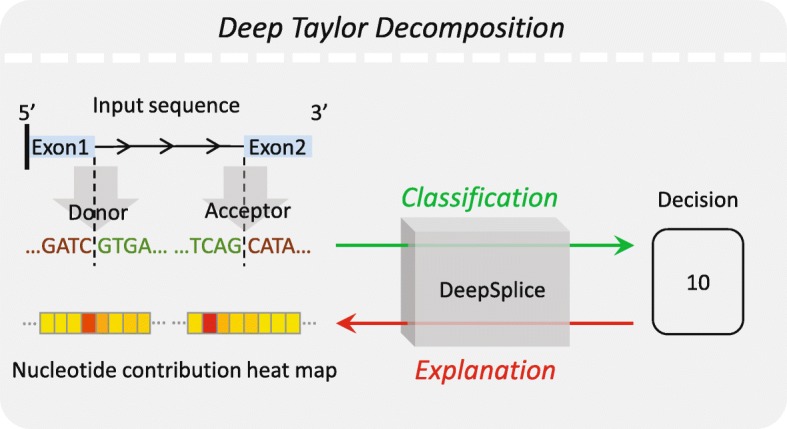
Visualization of deep Taylor decomposition in DeepSplice. Deep Taylor decomposition explains the contribution of each nucleotide in the splice junction sequence to the final decision function of the deep convolutional neural network. Deep Taylor decomposition operates by running a backward pass on the trained convolutional neural network using a predefined set of rules

We propose to use deep Taylor decomposition [40] to explain the contribution of nucleotides in the splice junction sequence to the final decision function of the deep convolutional neural network, as shown in Fig. 9. Taking image recognition task as an example, such decomposition results in a “heat map” that indicates what pixels of the image are important for a neural network classification. In our application, for testing splice junction sequence **S**, we would like to associate to nucleotide *n* a contribution score *C*_*n*_(**S**) from which it is possible to judge which nucleotides are of importance to explain the predicted label of class from the deep convolutional neural network.

Deep Taylor decomposition operates by running a backward pass on the trained convolutional neural network using a predefined set of rules. To decompose *C*_*j*_ on the set of lower layer neurons {*x*_*i*_} to which *x*_*j*_ is connected, Taylor decomposition [54] is employed, and *C*_*j*_ is given by:

2 $$ {\left.{C}_j={\left({\frac{\partial {C}_j}{\partial \left\{{x}_i\right\}}}_{{\left\{\overset{\sim }{x_i}\right\}}^{(j)}}\right)}^{\mathrm{T}}.\left(\left\{{x}_i\right\}-{\left\{\overset{\sim }{x_i}\right\}}^{(j)}\right)+{\varepsilon}_j=\sum \limits_i\frac{\partial {C}_j}{\partial {x}_i}\right|}_{{\left\{\overset{\sim }{x_i}\right\}}^{(j)}}.\left({x}_i-{\overset{\sim }{x_i}}^{(j)}\right)+{\varepsilon}_j=\sum \limits_i{C}_{ij}+{\varepsilon}_j, $$

where *ε*_*j*_ denotes the Taylor residual, and where $\mid {}_{{\left\{\overset{\sim }{x_i}\right\}}^{(j)}}$ indicates that the derivative has been evaluated at the root point ${\left\{\overset{\sim }{x_i}\right\}}^{(j)}$. The identified term *C*_*ij*_ is the redistributed contribution from neuron *x*_*j*_ to neuron *x*_*i*_ in the lower layers. To determine the total contribution of neuron *x*_*i*_, one needs to pool contribution coming from all neurons {*x*_*j*_} to which the neuron *x*_*i*_ contributes:

3 $$ {\left.{C}_i=\sum \limits_j{C}_{ij}=\sum \limits_j\frac{\partial {C}_j}{\partial {x}_i}\right|}_{{\left\{\overset{\sim }{x_i}\right\}}^{(j)}}.\left({x}_i-{\overset{\sim }{x_i}}^{(j)}\right), $$

which will be central for computing explicit contribution redistribution formulas based on specific choices of root points ${\left\{\overset{\sim }{x_i}\right\}}^{(j)}$. Backpropagating from the function output down to the input, it results in assigning a set of scores **C**(**S**) = {*C*_*n*_(**S**)} to the nucleotides in the input testing splice junction sequence **S** to quantify their contributions to the predicted label of class.

### Other deep learning architectures

To decipher the abilities of different deep learning architectures in handling splice junction sequence data, we further build multilayer perceptron network (MLP) and long short-term memory network (LSTM) to compare with convolutional neural network. MLP is a feedforward artificial neural network with multiple hidden layers of units between input and output layers. LSTM is a recurrent neural network architecture where connections between units form a directed cycle.

The multilayer perceptron network is composed of one input layer, four hidden layers and one output layer. Each layer is fully connected to next layer in the network. The number of neurons in each hidden layer is 64, 128, 128 and 256 respectively. In the long short-term memory network, we deploy one input layer, three hidden layers and one output layer. Each of the three hidden layers contains 16 LSTM cells. For both architectures, the inputs are splice junction sequences transformed by one-hot encoding, and the outputs are class labels. Advance deep learning techniques, dropout [52], regularization [51], mini-batch gradient descent [53] and Adam [50], are exploited in the supervised training steps in both networks.

### Filtering of false splice junction as a result of repetitive sequences

**Fig. 10:**
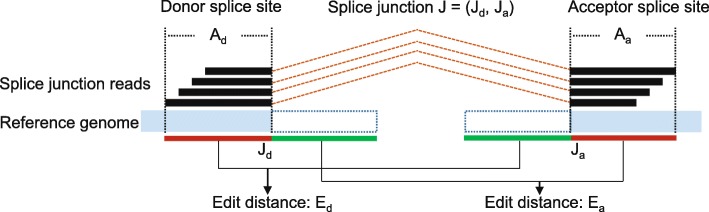
Illustration of splice junction filtering strategy. In this example, two edit distances are calculated. One (*E*_*d*_) is between anchor sequence at donor site (*G [J*_*d*_*-A*_*d*_ *+ 1:J*_*d*_*]*) and intermediate flanking sequence next to acceptor site (*G [J*_*a*_*-A*_*a*_*:J*_*a*_*-1]*). The other (*E*_*a*_) is between anchor sequence at acceptor site (*G [J*_*a*_*:J*_*a*_ *+ A*_*a*_*-1]*) and intermediate flanking sequence next to donor site (*G [J*_*d*_ *+ 1:J*_*d*_ *+ A*_*d*_*]*)

One potential resource of false positive splice junction is the inability to align a sequence to the correct sites due to higher mismatches than the threshold set by aligners or small indels that cannot be detected by aligners. Before the classification of splice junctions, we first remove the splice junctions whose sequence at the acceptor (donor) site has high sequence similarity with the immediate flanking sequence next to the donor (acceptor) site or the sequence at any of its alternative acceptor (donor) sites, as shown in Fig. 10. The edit distance between the alternative acceptor (donor) site sequences is computed using the Smith-Waterman algorithm [55]. This filtering strategy is independent of read coverage and enables the retention of correct splice junctions even with low read coverage. The removal of these sequences is necessary as most of them are highly similar with one of the splice junctions remaining in the data set.

### Implementation

The deep learning architectures are implemented using TensorFlow [56]. Training and testing are deployed on Nvidia GeForce GTX 1080 graphics cards. DeepSplice is freely available for academic use and can be accessible at https://github.com/zhangyimc/DeepSplice.

### Performance measures

We employ the following metrics: Area Under the ROC Curve (auROC), Area Under the Precision Recall Curve (auPRC), sensitivity, specificity, accuracy, F measure and *Q*^9^. *Q*^9^ is independent of the class distribution in the data set and is used to evaluate the classifier performance on splice site prediction [35], which is defined as: *Q*^9^ = (1 + *q*^9^)/2 where

4 $$ {q}^9=\left\{\begin{array}{c}\left( TN- FP\right)/\left( TN+ FP\right), if\  TP+ FN=0\\ {}\left( TP- FN\right)/\left( TP+ FN\right), if\  TN+ FP=0\\ {}1-\sqrt{2}\sqrt{\left[ FN/\right( TP+{FN}^2+{\left[ FP/\left( TN+ FP\right)\right]}^2}, if\  TP+ FN\ne 0\  and\  TN+ FP\ne 0\end{array}.\right. $$

## Additional file

Additional file 1: **Figures S1**, **S2**, **S3**, **S4** and **S5**. **Table S1** and **S2**. (PDF 1105 kb)

## Abbreviations

SRA: Sequence Read Archive
HS3D: *Homo Sapiens* Splice Sites Database
CNN: convolutional neural network
ConvNet: convolutional neural network
MLP: multilayer perceptron network
LSTM: long short-term memory network
auROC: Area Under the ROC Curve
auPRC: Area Under the Precision Recall Curve
