# Supplementary material for: Discerning novel splice junctions derived from RNA-seq alignment: a deep learning approach
Source: BMC Genomics. 2018 Dec 27;19:971. doi: 10.1186/s12864-018-5350-1 (PMC6307148; doi:10.1186/s12864-018-5350-1)
Supplement: Supplementary file 1 — Figures S1, S2, S3, S4 and S5. Table S1 and S2. (PDF 1105 kb) [file 12864_2018_5350_MOESM1_ESM.pdf]

Supplementary Information

Submitted along with "Discerning novel splice junctions derived from RNA-seq alignment: a deep learning approach", Yi Zhang[1], Xinan Liu[1], James MacLeod[2], Jinze Liu[1]

[1] Department of Computer Science, University of Kentucky, Lexington, KY 40506, USA

[2] Department of Veterinary Science, University of Kentucky, Lexington, KY 40506, USA

SUPPLEMENTARY FIGURES

Figure S1. (a) Discrete proportions of negatives, positive splice junctions without annotated site, positive splice junctions with acceptor site annotated, positive splice junctions with donor site annotated and positive splice junctions with two sides annotated, given the total read support. (b) Cumulative proportions of positive splice junctions in each category with the increase of the total read support.

(a)

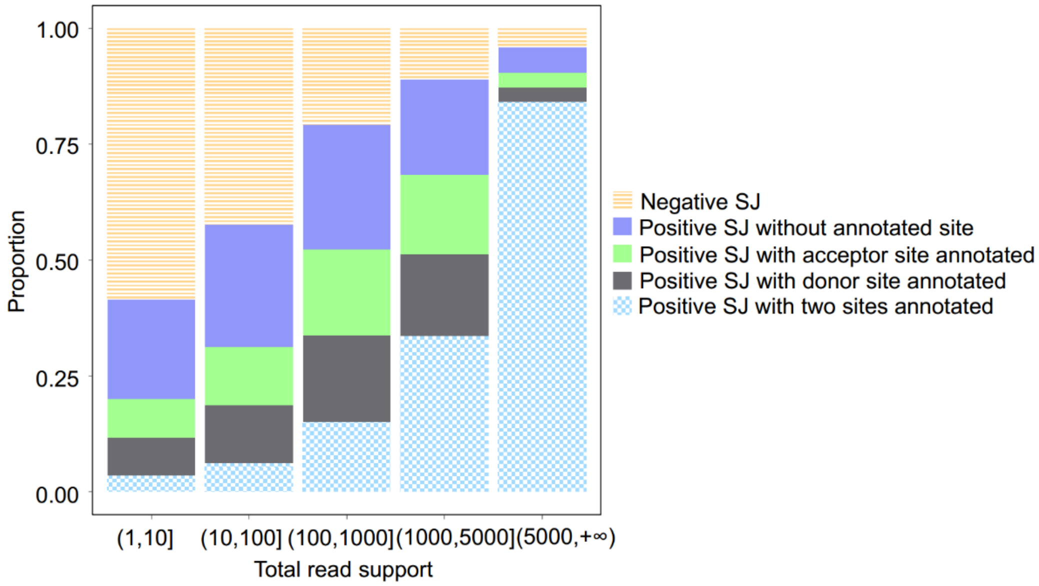

(b)

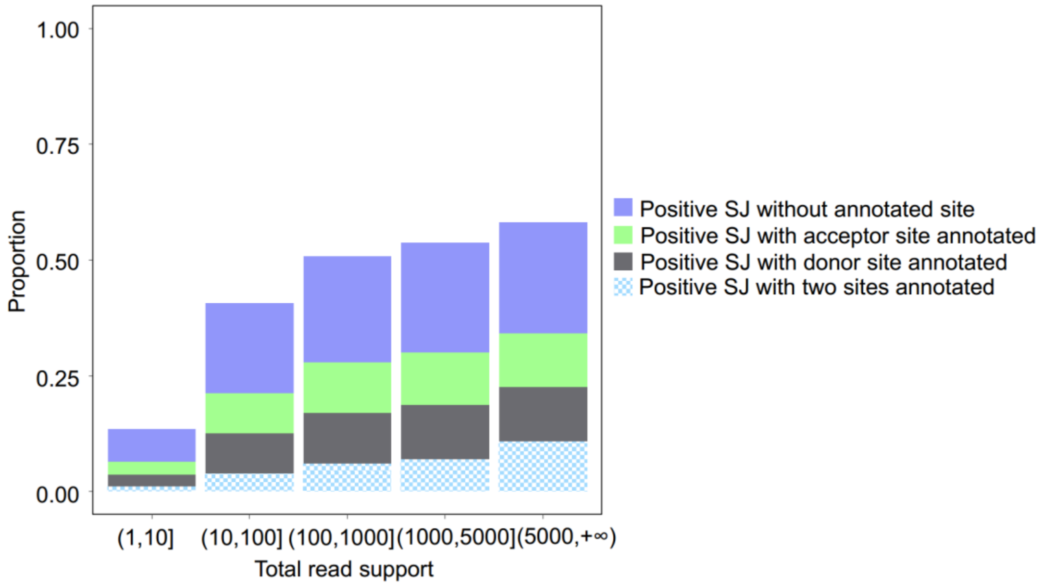

Figure S2. (a) Discrete proportions of negatives, positive semi-canonical splice junctions and positive canonical splice junctions from the classification results, given the total read support. (b) Cumulative proportions of positive semi-canonical and canonical splice junctions with the increase of the total read support.

(a)

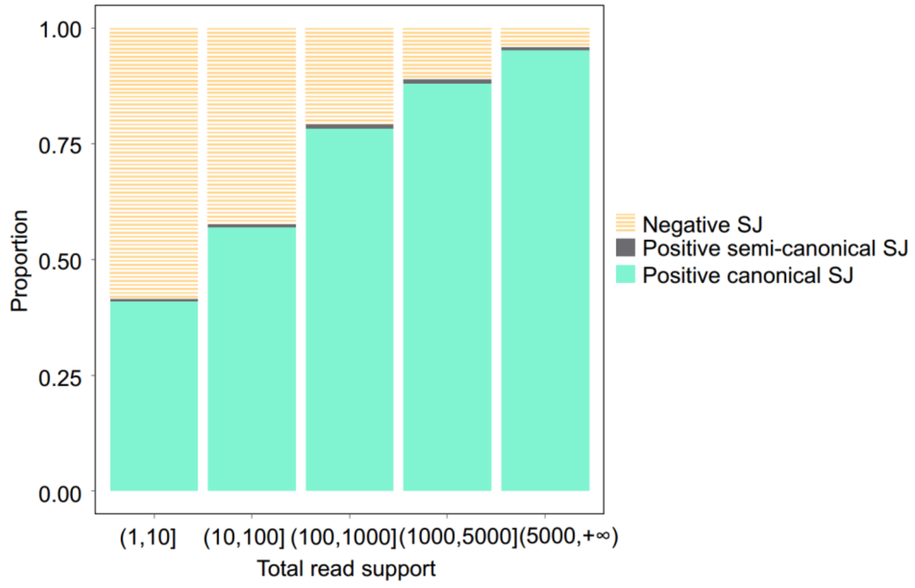

(b)

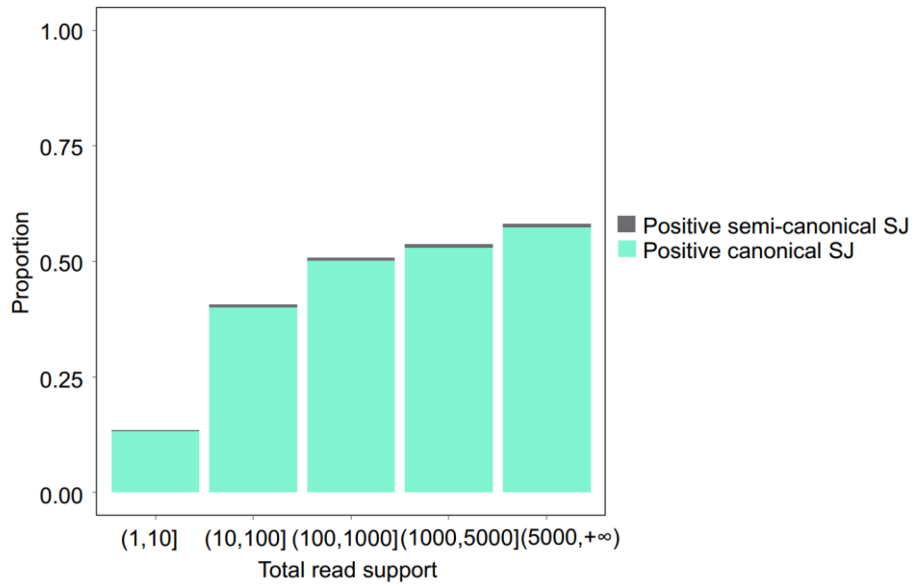

Figure S3. (a) Discrete proportions of negatives, positive splice junctions without annotated site, positive splice junctions with acceptor site annotated, positive splice junctions with donor site annotated and positive splice junctions with two sides annotated, given the reoccurrence in samples. (b) Cumulative proportions of positive splice junctions in each category with the increase of the reoccurrence in samples.

(a)

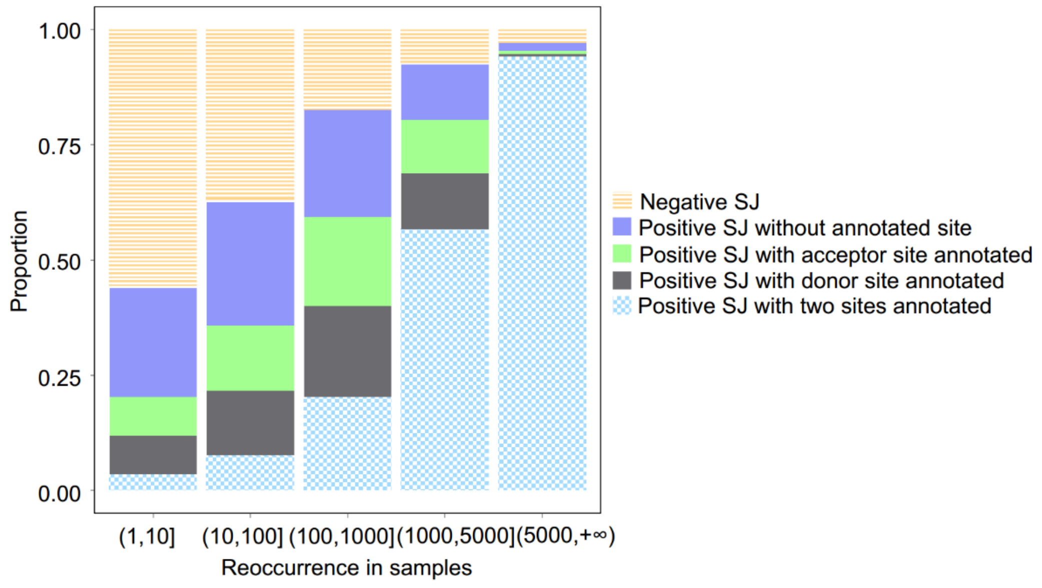

(b)

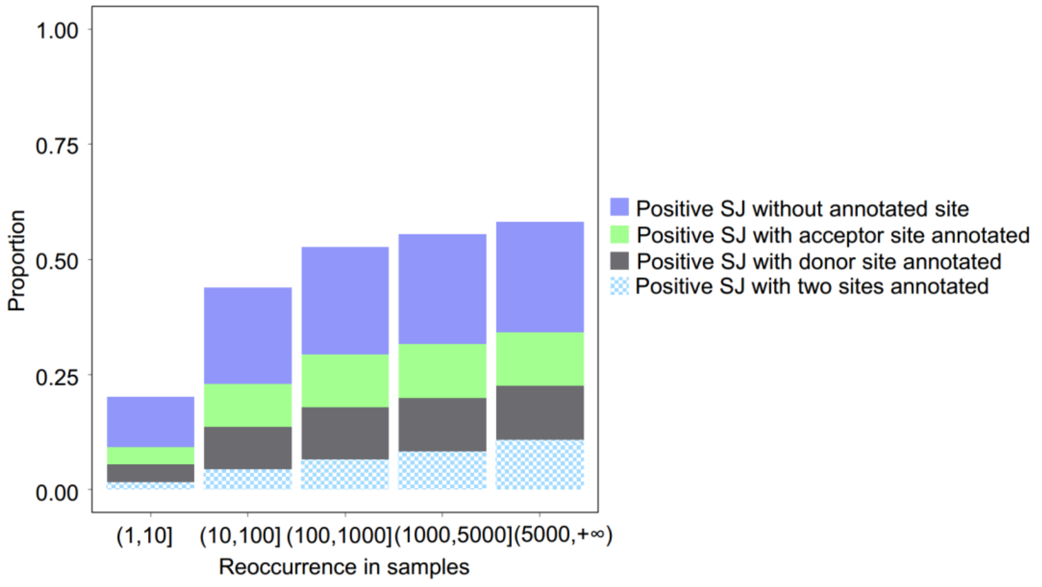

Figure S4. (a) Discrete proportions of negatives, positive semi-canonical splice junctions and positive canonical splice junctions from the classification results, given the reoccurrence in samples. (b) Cumulative proportions of positive semi-canonical and canonical splice junctions with the increase of the reoccurrence in samples.

(a)

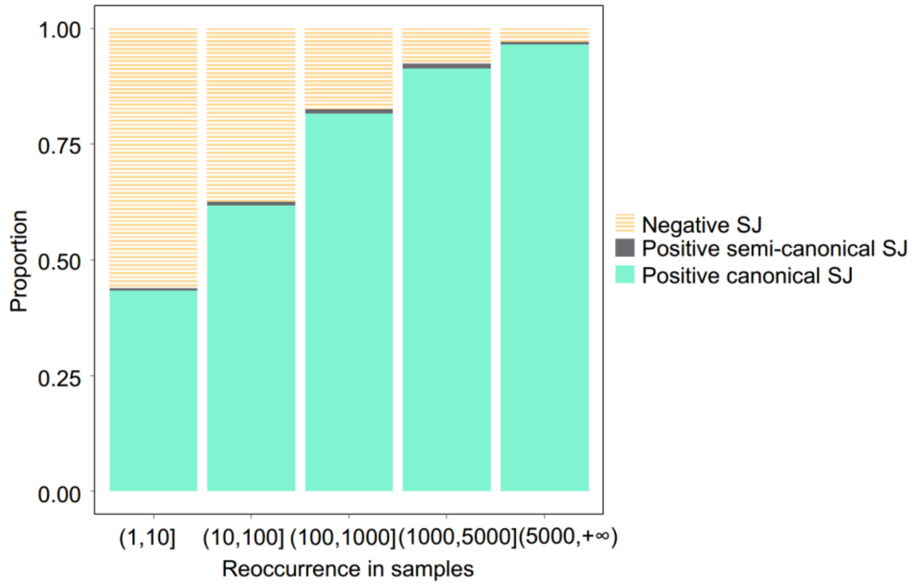

(b)

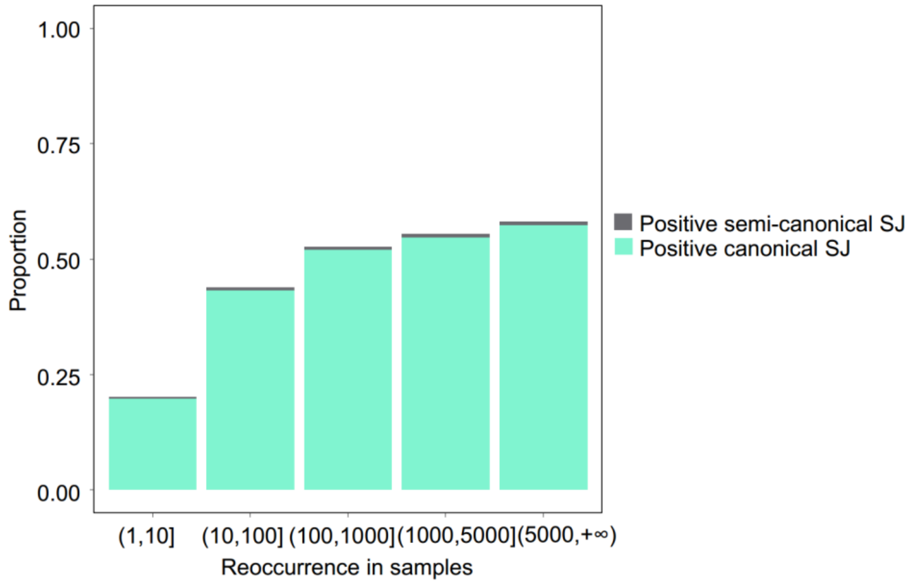

Figure S5. Visualization of the information content in bits[1] of each type of nucleotide in the flanking splice sequence for splice junctions classified as positives by DeepSplice.

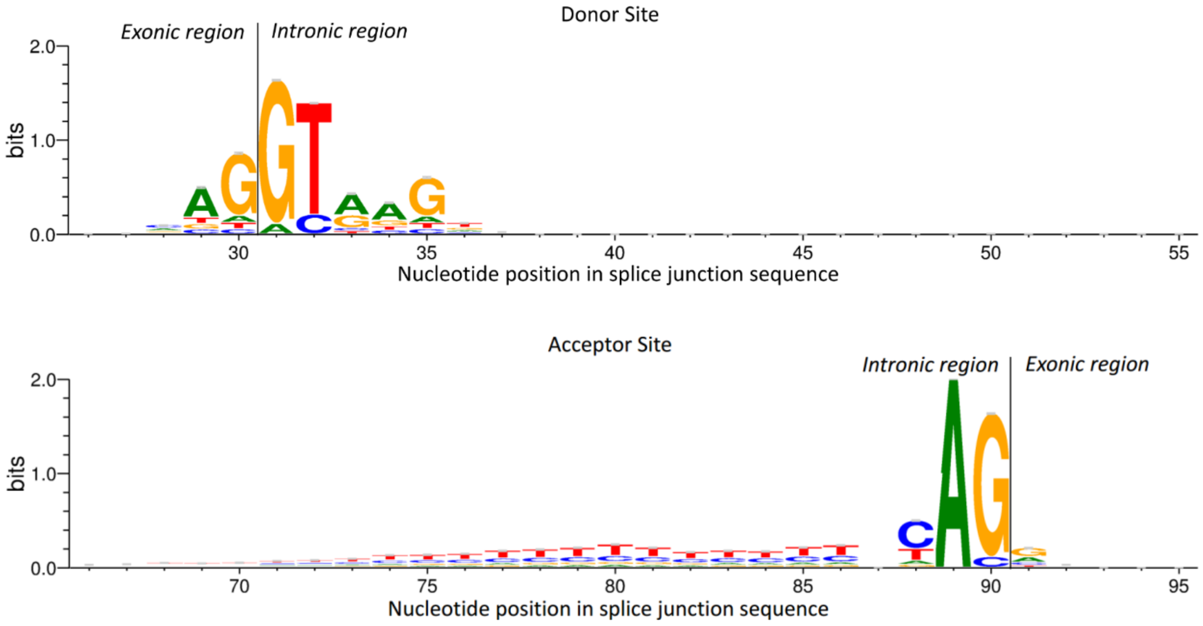

SUPPLEMENTARY TABLES

Table S1. Comparison of classification accuracy between models varying splice junction sequence length, number of convolutional layers, and learning rate. Through increasing the length of genomic sequences from 10 nucleotides to 40 nucleotides, a consistent increase in accuracy is observed. The increase of genomic sequence length over 30 nucleotides results in minimal further improvement of classification performance. Deep convolutional neural networks with two convolutional layers outperformed those with one convolutional layer in terms of accuracy. Models trained with learning rate of 0.001 achieved better performance than others. To evaluate the performance of models with different parameter settings, 10-fold cross-validation was used.

Sequence Length	Number of Convolutional Layer	Learning Rate	Accuracy
10	One	0.01	0.929
20	One	0.01	0.937
30	One	0.01	0.942
40	One	0.01	0.942
10	Two	0.01	0.934
20	Two	0.01	0.944
30	Two	0.01	0.948
40	Two	0.01	0.949
10	One	0.001	0.938
20	One	0.001	0.945
30	One	0.001	0.951
40	One	0.001	0.951
10	Two	0.001	0.942
20	Two	0.001	0.953
30	Two	0.001	0.956
40	Two	0.001	0.957
10	One	0.0001	0.936
20	One	0.0001	0.943
30	One	0.0001	0.950
40	One	0.0001	0.950
10	Two	0.0001	0.940
20	Two	0.0001	0.951
30	Two	0.0001	0.955
40	Two	0.0001	0.956

Table S2. Classification performance evaluation of DeepSplice and an out-of-the-box convolutional neural network without functional pairing (OOTB_CNN) on GENCODE data set.

	Sensitivity	*Specificity*	*Accuracy*	*F1 score*
DeepSplice	0.943	0.968	0.956	0.955
OOTB_CNN	0.729	0.966	0.847	0.827

REFERENCES

1 Crooks, G. E., Hon, G., Chandonia, J.-M. & Brenner, S. E. WebLogo: a sequence logo generator. *Genome research* **14**, 1188-1190 (2004).
